# Cutaneous microvascular vasodilatory consequences of acute consumption of a caffeinated soft drink sweetened with high‐fructose corn syrup

**DOI:** 10.14814/phy2.15074

**Published:** 2021-10-21

**Authors:** Joel T. Greenshields, Jason M. Keeler, Jessica A. Freemas, Tyler B. Baker, Blair D. Johnson, Stephen J. Carter, Zachary J. Schlader

**Affiliations:** ^1^ H.H. Morris Human Performance Laboratories Department of Kinesiology School of Public Health Indiana University Bloomington Indiana USA; ^2^ Cancer Prevention and Control Program Indiana University Melvin and Bren Simon Comprehensive Cancer Center Indianapolis Indiana USA

**Keywords:** high‐fructose corn syrup, ischemia‐reperfusion injury, local skin heating, microvasculature, soft drink

## Abstract

This study tested the hypotheses that compared to drinking water, consumption of a caffeinated soft drink sweetened with high‐fructose corn syrup (HFCS) attenuates the cutaneous vasodilatory response to local skin heating without (Protocol 1) and following ischemia‐reperfusion injury (Protocol 2). In a randomized, counterbalanced crossover design, 14 healthy adults (25 ± 3 year, 6 women) consumed 500 ml of water (water) or a caffeinated soft drink sweetened with HFCS (Mtn. Dew, DEW). Thirty minutes following beverage consumption local skin heating commenced on the right forearm (Protocol 1), while on the left forearm ischemia‐reperfusion commenced with 20 min of ischemia followed by 20 min of reperfusion and then local skin heating (Protocol 2). Local skin heating involved 40 min of heating to 39℃ followed by 20 min of heating to 44℃. Skin blood flow (SkBf, laser Doppler) data were normalized to mean arterial pressure and are presented as a cutaneous vascular conductance (CVC) and as percentage of the CVC response during heating to 44℃ (%CVC_max_). Protocol 1: During local heating at 39℃, no differences were observed in CVC (water: 2.0 ± 0.6 PU/mmHg; DEW: 2.0 ± 0.8 PU/mmHg, *p* = 0.83) or %CVC_max_ (water: 59 ± 14%; DEW 60 ± 15%, *p* = 0.84) between trials. Protocol 2: During local skin heating at 39℃, no differences were observed in CVC (water: 1.7 ± 0.5 PU/mmHg; DEW: 1.5 ± 0.5 PU/mmHg, *p *= 0.33) or %CVC_max_ (water: 64 ± 15%; DEW 61 ± 15% *p* = 0.62) between trials. The cutaneous microvascular vasodilator response to local heating with or without prior ischemia‐reperfusion injury is not affected by acute consumption of a caffeinated soft drink sweetened with HFCS.

## Graphical abstract

We examined the effect of consumption of a caffeinated soft drink sweetened with high‐fructose corn syrup on the cutaneous vasodilatory response to local skin heating without and following ischemia‐reperfusion injury. We identified that the cutaneous microvascular vasodilator response to local heating with or without prior ischemia‐reperfusion injury is not affected by acute consumption of a caffeinated soft drink sweetened with high‐fructose corn syrup.

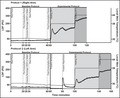

## 1 INTRODUCTION

Excessive habitual consumption of soft drinks sweetened with high‐fructose corn syrup (HFCS) is associated with a heightened risk of cardiovascular disease (Malik & Hu, 2015). Fructose consumption elevates blood pressure likely by increasing cardiac output without compensatory reductions in vascular resistance (Brown et al., 2008). This is supported by mechanistic evidence that fructose consumption shifts the vascular vasodilator–vasoconstrictor balance toward vasoconstriction (Wang et al., 2006). Previous work has identified that consuming Mountain Dew®, a caffeinated soft drink sweetened with HFCS, augments the renal vasoconstrictor response during sympathetic stimulation compared to water and other soft drinks matched for caffeine content (Chapman et al., 2020). It is unclear whether the observed vascular effects are specific to the kidneys or indicative of a systemic vascular response. For instance, consumption of a caffeinated soft drink sweetened with HFCS decreases cardiovagal baroreflex sensitivity, likely due to modulation of the parasympathetic nervous system (Chapman et al., 2021). However, consumption of this same beverage did not affect arterial stiffness, likely due to minimal effect on sympathetic activation (Freemas et al., 2021). Given the association between high intake of soft drinks containing HFCS and cardiovascular disease (Malik & Hu, 2015), it is possible that these findings reflect HFCS‐medicated changes throughout the vascular tree (i.e., not only in the renal vasculature).

Cardiovascular disease is often first manifested in the tissue microcirculation (Minson, 2010). Microvascular endothelial dysfunction is characterized by a shift toward vasoconstrictor tone attributed to reductions in nitric oxide bioavailability, but also contributed to by other vasoconstrictor responses (e.g., sympathetic activity) (Amiya et al., 2014; Choi et al., 2014; Holowatz et al., 2008). Notably, microvascular endothelial dysfunction is one of the earliest pathogenic vascular changes leading to cardiovascular disease (Holowatz et al., 2008). Thus, examination of microvascular endothelial function following ingestion of a caffeinated soft drink sweetened with HFCS may provide unique insights toward understanding the mechanisms by which consumption of these beverages may increase the risk of cardiovascular disease. Local heating of the cutaneous vasculature is often used as a model to examine generalized microvascular function, which is at least partially related to endothelium‐dependent mechanisms (Holowatz et al., 2008; Minson, 2010). Therefore, the primary purpose of this study (*Protocol 1)* was to test the hypothesis that compared to drinking water, consumption of a caffeinated soft drink sweetened with HFCS (Mountain Dew) attenuates the cutaneous vasodilatory response to local skin heating, a primarily nitric oxide‐mediated response (Choi et al., 2014).

Ischemia‐reperfusion injury is a primary cause of poor outcomes following ischemic cardiac events (Turer & Hill, 2010). Moreover, ischemia‐reperfusion injury of the forearm in healthy humans blunts the cutaneous vasodilatory response to local heating on that forearm, likely due to impairments in microvascular endothelial function (McGarr et al., 2018). Since ischemia‐reperfusion is a known insult to the vasodilatory response, we suspect that the addition of a caffeinated soft drink sweetened with HFCS could exacerbate impairments of microvascular endothelial function through disruptions in nitric oxide bioavailability. Thus, a secondary purpose of this study (*Protocol 2)* was to test the hypothesis that the cutaneous vasodilatory response to local skin heating following ischemia‐reperfusion injury is attenuated following acute consumption of a caffeinated soft drink sweetened with HFCS (Mountain Dew) compared to water.

## 2 METHODS

### 2.1 Participants

An a priori power analysis was performed with G‐Power version 3.1.9.4 using data from our laboratory demonstrating that Mountain Dew sweetened with HFCS increased renal vascular resistance by 0.6 ± 0.7 mmHg/cm/s (Chapman et al., 2020) (Cohen's *d*_z_ = 0.86). Using this effect size, we estimated that at least 13 participants were needed to detect differences in skin blood flow using standard parameters of 1‐β = 0.80 and α = 0.05. Therefore, for a counterbalanced design, 14 healthy adults (25 ± 3 year, six women) completed both study protocols. Participant characteristics were––height: 173 ± 9 cm, body mass: 73.5 ± 13.9 kg, body mass index: 24.6 ± 3.6 kg/m^2^, systolic blood pressure 123 ± 5 mmHg, and diastolic blood pressure 79 ± 6 mmHg. Participants provided written consent after being fully informed of the experimental procedures and possible risks. Participants self‐reported to be physically active, nonsmokers, and reported to be free from any cardiovascular, metabolic, renal, or neurological diseases. Female participants self‐reported to be normally menstruating, were confirmed to not be pregnant via a urine pregnancy test, and were tested in the first 10 days following menstruation (mean ± SD, 5 ± 3 days). Participants self‐reported to regularly consume caffeinated soft drinks 1 ± 2 days per week, but neither the inclusion nor exclusion criteria for this study considered habitual sugar‐sweetened soft drink consumption. Both study protocols were approved by the Institutional Review Board at Indiana University and performed in accordance with the standards set by the latest revision of the Declaration of Helsinki, except for registration in a database. These data were collected concurrently with those presented in a previously published manuscript that tested a unique research hypothesis related to the effects of ingesting a commercially available caffeinated soft drink sweetened with HFCS on central hemodynamics (Freemas et al., 2021).

### 2.2 Instrumentation and Measurements

Body mass (kg) and height (cm) were measured using a stadiometer (Holtain Limited, Seritex, Wales, UK) and scale (Sauter, Balingen, Germany). Urine‐specific gravity was measured using a refractometer (Atago, Tokyo, Japan). Heart rate (bpm) was continually measured via a 3‐lead ECG (Datex‐Ohmeda, Helsinki, Finland). Pulse oximetry (Nellcor N600x, Medtronic Inc. USA) was used to estimate arterial oxygen saturation (S_p_O_2_, %) and was measured at the finger on both the left and right hands. Beat‐by‐beat blood pressure was measured using the Penaz method (NIBP Nano, AD Instruments, Colorado Springs, CO, USA) on the right hand. Beat‐to beat‐blood pressure data were height corrected and corrected to manual brachial artery auscultatory measures taken in duplicate.

Local skin heaters (Moor Instruments, Devon, UK) covering an area of 11 mm^2^ were placed on two sites of the right dorsal forearm, and one on the dorsal portion of the left forearm. Red blood cell flux, an index of skin blood flow (SkBf, PU), was measured at each site using integrated laser Doppler flowmetry probes (Moor Instruments, Devon, UK), which were seated in the center of each local heater. Local heater and laser Doppler flowmetry probe placement were maintained for the second trial by marking the circumference of the local heater on the forearm in semi‐permanent ink during the first trial. Local forearm skin temperatures (T_skin_, °C) were measured via temperature probes imbedded in the local heating units (Moor Instruments, Devon, UK).

#### 2.2.1 Overview

During two experimental visits, subjects rested comfortably in the supine position. In a randomized counterbalanced crossover design, participants consumed either water (Water, Dasani®, The Coca Cola Company) or a caffeinated soft drink sweetened with HFCS (DEW, Mountain Dew®, Pepsi Co). Data were converted from analog to digital with a 16‐channel data acquisition system (PowerLab 16/35, ADInstruments) sampling at 1000 Hz, and were collected using LabChart software version 8.1.16 (ADInstruments).

#### 2.2.2 Pre‐500 ml fluid consumption

**FIGURE 1:**
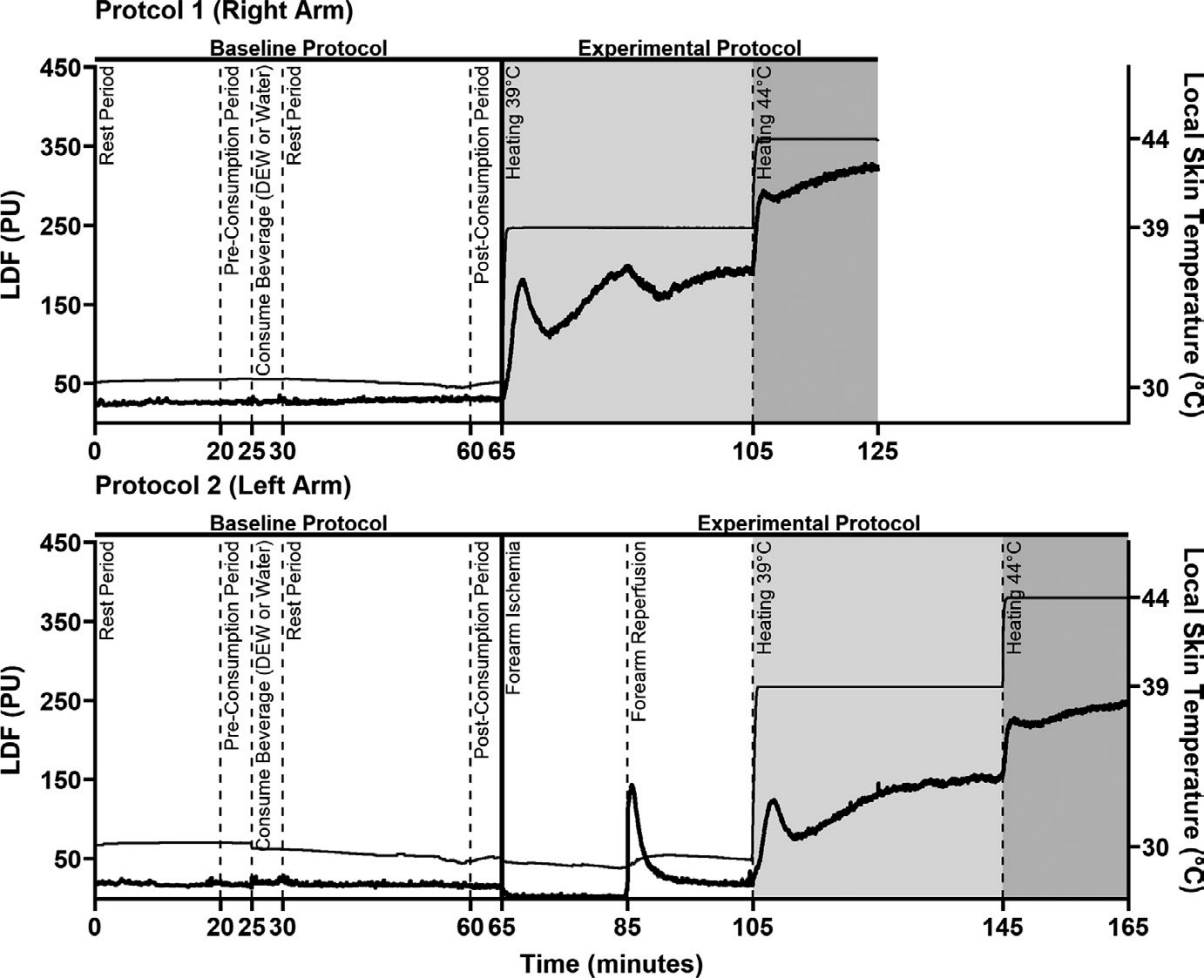
Study timeline and amalgamated skin blood flow data. Both protocols went through the same baseline protocol that consisted of pre‐consumption baseline, a 5‐min beverage consumption period, and a post‐consumption baseline that was preceded by 30 min of supine rest. Following the baseline protocol, Protocol 1 (right arm) started with local skin heating at 39℃ for 40 min followed by 20 min of local skin heating to 44℃. Meanwhile, Protocol 2 (left arm) underwent 20 min of ischemia, then 20 min of reperfusion, followed by the same local skin heating design of 40 min at 39℃ followed by 20 min of local skin heating to 44℃. Note the unexpected acute reduction in skin blood flow on the right forearm that coincides with the onset of forearm reperfusion on the left arm. This experimental consideration has been thoroughly discussed in the text of the discussion

Participants reported to the temperature‐controlled laboratory (DEW: 23 ± 1℃, 25 ± 6% relative humidity; water 23 ± 1℃, 24 ± 5% relative humidity) after abstaining from exercise, caffeine, and alcohol for 12 hr, and food for 2 hr. The ambient temperature and relative humidity were not significantly different between trials (paired *t*‐test, *p* ≥ 0.51). The second experimental visit took place at least 48 hr after the first visit, and participants arrived at the laboratory at the same time of day as their first visit to control for any potential diurnal effects. Upon arrival, participants provided a urine sample. Euhydration was confirmed at this time via a urine‐specific gravity <1.020. After instrumentation and a 20‐min rest period in which participants laid supine, a 5‐min pre‐500 ml fluid consumption baseline (pre‐consumption) was established (Figure 1).

#### 2.2.3 Post‐500 ml fluid consumption

Following the pre‐consumption period, participants were given 5 min to consume 500 ml of water or DEW chilled to about ~11℃, and then resumed the supine position (Figure 1). Mountain Dew is a high calorie, high‐fructose, caffeinated soft drink that has previously been demonstrated to cause renal vasoconstriction during rest and sympathetic activation (Chapman et al., 2020) and reduce spontaneous cardiovagal baroreflex sensitivity (Chapman et al., 2021). Mountain Dew has one of the highest free fructose concentrations among commercially available soft drinks, with fructose and glucose each representing 59.5% and 39.7%, respectively, of the total sugar content of the beverage (Walker et al., 2014). According to an analysis from Walker et al., ~36 g of fructose and ~25 g of glucose are present in 500 ml of Mountain Dew (Walker et al., 2014). After ingesting 500 ml of the assigned beverage within 5 min, participants rested supine for 30 min. At 30‐min post‐consumption, a 5‐min post‐consumption baseline (post‐consumption) was established. Testing was completed 30‐min post‐fluid consumption, because previous data shows that increases in fructose in the blood following oral ingestion can be detected at this time, thus ensuring that the high‐fructose soft drink was in circulation (Le et al., 2012). After this 30‐min post‐fluid consumption data collection period, two protocols commenced––Protocol 1 (right arm) and Protocol 2 (left arm) (Figure 1). Throughout the pre‐ and post‐consumption data collection periods the local heaters were turned off, permitting the measurement of T_skin_.

##### Protocol 1––Effect of high‐fructose corn syrup sweetened caffeinated soft drink consumption on the vasodilatory response during local heating (right arm)

Following the pre‐ and post‐consumption periods, subjects continued to rest supine, and local heating at 39℃ to the two locations on the right forearm began for 40 min. Heating at 39℃ was chosen because at this skin temperature and duration of heating the plateau skin blood flow response is primarily (~80%) nitric oxide‐dependent (Choi et al., 2014). Following the 40‐min period at 39℃, the temperature was increased to 44℃ for 20 min to elicit a local heating‐induced maximal vasodilatory response (Choi et al., 2014). Local heating was applied at a rate of 0.3℃ s^−1^. By design, the laser Doppler flowmetry data in Protocol 1 were combined and presented as an average between the two probes to improve the spatial resolution of the measurement. This decision was supported by post hoc analyses that indicated that the overall estimated difference between the two probes on the right forearm was 1 PU (95% CI: −16 to 19 PU), and there were no differences during any of the experimental time points.

##### Protocol 2––Effect of high‐fructose corn syrup sweetened caffeinated soft drink consumption on the vasodilatory response during local heating following ischemia‐reperfusion (left arm)

On the left arm, following the post‐consumption baseline data collection period, participants underwent 20 min of experimental ischemia, administered by a cuff wrapped around the upper arm and held constant at a pressure of 220 mmHg (E20 Rapid Cuff Inflator, Hokanson Inc., Bellevue, WA, USA). After 20 min, the cuff was released and participants rested during a 20‐minute reperfusion period, following which skin on left forearm was heated locally to a temperature of 39℃ for 40 min and then 44℃ for 20 min (McGarr et al., 2018). Laser Doppler flowmetry data in Protocol 2 were collected at two different sites, glabrous skin (fingertip) and non‐glabrous skin (forearm) based on previous findings demonstrating differences in cutaneous microvascular function following ischemia‐reperfusion between these two skin types (McGarr et al., 2018). However, upon post hoc inspection of the fingertip skin blood flow data, it was determined that these data were unreliable and inaccurate, likely due to poor adhesion of the laser Doppler probe to the skin of the fingertip. Thus, these data are not presented in this investigation.

The timing of the forearm ischemia‐reperfusion injury (Protocol 2) was chosen to not influence the local heating responses on the right arm (Protocol 1) (Figure 1). Specifically, ischemia‐reperfusion injury increases sympathetic nerve activity (Lambert et al., 2016; Loukogeorgakis et al., 2005), which can reduce endothelial‐dependent vasodilation (Lambert et al., 2016; Loukogeorgakis et al., 2005). However, the increases in muscle sympathetic nerve activity during the latter stages of ischemia and initial period of reperfusion have abated by the late stages of reperfusion (Lambert et al., 2016), which coincides with the latter portion of the local heating to 39℃ procedures on the right arm (Protocol 1). Thus, it is unlikely that ischemia‐reperfusion injury on the left arm (Protocol 2) influenced the skin blood flow responses on the right arm (Protocol 1). If it did, however, it is important to note that this effect would have been the same in both the water and DEW trials. Therefore, we are confident that this split body approach allowed us to test our research hypotheses, while minimizing participant burden.

### 2.3 Data and statistical analyses

Prior to data aggregation for analysis, data that were collected during heating at 39℃ and 44℃ were visually inspected to ensure that a plateau in SkBf was observed, which was the case in all instances. Data were averaged across the final 5 min during the pre‐ and post‐consumption periods, and during heating at 39℃ and 44℃ to be used in statistical analyses. Cutaneous vascular conductance (CVC, PU/mmHg) was calculated as SkBf divided by the mean arterial pressure. SkBf and CVC were then represented as a percentage of maximum local heating‐induced vasodilatory response as identified during heating at 44℃ (Chaseling et al., 2020) (%SkBf_max_ and %CVC_max_). Furthermore, initial peak SkBf and initial peak CVC were calculated by averaging the values over a 30‐s period and were expressed as local maximum of SkBF or CVC during heating at 44℃.

During Protocol 2, the 20‐min reperfusion period data were analyzed to determine the reactive hyperemia response. Peak SkBf (PU) during reperfusion, time to SkBf_max_ (seconds), and peak skin blood flow as a percentage of skin blood flow during heating at 44℃ (%SkBf_max_, %) were calculated during the initial reperfusion period. Additionally, SkBf (PU min) and %SkBf_max_ (% min) were integrated during the 20‐min reperfusion period to calculate area under the curve above baseline prior to ischemia taken over a 5‐min period (5.5 min to 30 s prior to ischemia to remove any movement artifact causes by cuff placement).

Prior to analysis, normality of data and the presence of outliers were evaluated using normal Quantile–Quantile (Q–Q) and kernel density plots, and no outliers were identified. Pre‐ and post‐consumption data were analyzed using a two‐way repeated measures ANOVA (time: pre‐ or post‐consumption, trial: water or DEW, and the interaction between time x trial) and, when a significant main effect or interaction was observed post hoc tests using Sidak's method for multiple corrections were conducted (Tybout & Sternthal, 2001). Following model fit, the assumptions of homoscedasticity and normality of the residuals necessary for the repeated measures ANOVA were checked, and no corrections were necessary. Data collected during local heating at 39℃ and 44℃, during initial peak analysis, and reactive hyperemia were analyzed using paired samples *t*‐tests. Prior to testing, normality in the paired pre‐ and post‐consumption values was checked using normal Q–Q plots and using the Shapiro–Wilk's test for normality, and no corrections were necessary. Study protocols were treated as independent studies that tested separate hypotheses. Therefore, no comparisons were made between the results from Protocol 1 and Protocol 2. Additionally, any comparisons between study protocols were not possible due to differences in measurement timing post‐beverage consumption. Data were analyzed using GraphPad Prism Version 9.1.2 (GraphPad Software Inc) and R version 4.1.0 (R Foundation for Statistical Computing). A priori statistical significance was set at *p* ≤ 0.05, and actual *p*‐values are reported where possible, along with 95% confidence intervals (95% CIs) when significant differences were identified. Data are reported as mean ±SD.

## 3 RESULTS

### 3.1 Resting physiological responses to 500 ml fluid consumption

Group pre‐ and post‐consumption data are presented in Table 1. There were no differences between the DEW or water trials in SkBf, %SkBf_max_, CVC, %CVC_max_, S_p_O_2_, or T_skin_ (see Table 1 for *p*‐values from repeated measures ANOVA). An effect of time (pre‐ vs. post‐consumption) was found for mean arterial pressure (*p* = 0.01), post hoc tests revealed a difference in DEW (4 ± 5 mmHg, 95% CI 0 to 8, *p* = 0.04), but not water (4 ± 7 mmHg, 95% CI −1 to 9 mmHg, *p* = 0.09). Heart rate showed a significant effect for trial (*p* = 0.04), time (*p* < 0.01), but not the interaction (*p* = 0.58). Post hoc tests showed no differences pre‐consumption (*p* = 0.20) or post‐consumption (*p* = 0.07) between the water and DEW trials; within‐trial differences were observed from pre‐consumption to post‐consumption in both the DEW (−3 ± 4 bpm, 95% CI −6 to 0 bpm, *p* = 0.04) and water (−4 ± 2 bpm, 95% CI −5 to −2 bpm, *p* < 0.01) trials.

**TABLE 1:** Pre‐ and Post‐500 ml fluid consumption

	Water		DEW		*P*‐value		
	Pre	Post	Pre	Post	Time	Trial	Time x Trial
MAP (mmHg)	91 ± 7	96 ± 8	92 ± 8	96 ± 9	0.01	0.82	0.82
HR (bpm)	56 ± 6	52 ± 6	58 ± 9	55 ± 8	<0.01	0.04	0.58
S_p_O_2_ (%)	98 ± 1	98 ± 1	98 ± 1	98 ± 1	0.19	0.39	0.78
T_skin_ (°C)	30.1 ± 1.7	29.7 ± 0.8	30.2 ± 0.9	29.8 ± 1.0	0.12	0.84	0.89
Protocol 1							
Right forearm							
SkBf (PU)	27 ± 16	28 ± 22	25 ± 16	33 ± 38	0.42	0.79	0.50
%SkBf_max_ (%)	8 ± 5	8 ± 6	8 ± 5	11 ± 12	0.46	0.66	0.48
CVC (PU/mmHg)	0.3 ± 0.2	0.3 ± 0.3	0.3 ± 0.2	0.4 ± 0.4	0.51	0.87	0.53
%CVC_max_ (%)	9 ± 5	9 ± 7	9 ± 7	12 ± 14	0.60	0.59	0.52
Protocol 2							
Left forearm							
SkBf (PU)	19 ± 14	17 ± 11	16 ± 11	13 ± 8	0.28	0.25	0.83
%SkBf_max_ (%)	8 ± 6	7 ± 4	7 ± 5	6 ± 4	0.27	0.44	0.75
CVC(PU/mmHg)	0.2 ± 0.1	0.2 ± 0.1	0.2 ± 0.1	0.1 ± 0.1	0.15	0.22	0.81
%CVC_max_ (%)	8 ± 6	8 ± 6	8 ± 7	6 ± 4	0.20	0.57	0.60

Note

Mean ±standard deviation.

*P*‐values presented from *F*‐tests for the main effects and interaction from repeated measures ANOVA.

Abbreviations: CVC, cutaneous vascular conductance, SkBf, skin blood flow, MAP, mean arterial pressure, PU, perfusion units (red blood cell flux), T_skin_, local forearm skin temperature.

#### 3.1.1 *Protocol 1––*Effect of high‐fructose corn syrup sweetened caffeinated soft drink consumption on the vasodilatory response during local heating

Data during local heating at 44℃ on the right arm are presented in Table 2. At 44℃, no differences in SkBf (*p* = 0.96) or CVC (*p* = 0.86) were identified between the water or DEW trial. With local heating at 44℃, heart rate was higher during the DEW trial (4 ± 5 bpm, 95% CI: 2–7 bpm, *p* < 0.01) compared to the water trial. Mean arterial pressure (*p* = 0.76) and S_p_O_2_ (*p* = 0.68) were not different between trials.

**TABLE 2:** Protocol 1 (right arm) Heating at 44℃ following 500 ml fluid consumption

	Water	DEW	*P*‐value
SkBf (PU)	322 ± 59	321 ± 66	0.96
CVC (PU/mmHg)	3.3 ± 0.7	3.3 ± 0.9	0.85
MAP (mmHg)	99 ± 12	100 ± 13	0.76
HR (bpm)	55 ± 7	60 ± 8	0.01
S_p_O_2_ (%)	97± 1	97 ± 1	0.68

Note

Mean ±standard deviation.

*P*‐values from paired samples *t*‐tests are presented.

Abbreviation: SkBf, skin blood flow, CVC, cutaneous vascular conductance, PU, perfusion units (red blood cell flux), MAP, mean arterial pressure, HR, heart rate, S_p_O_2_ arterial oxygen saturation.

**FIGURE 2:**
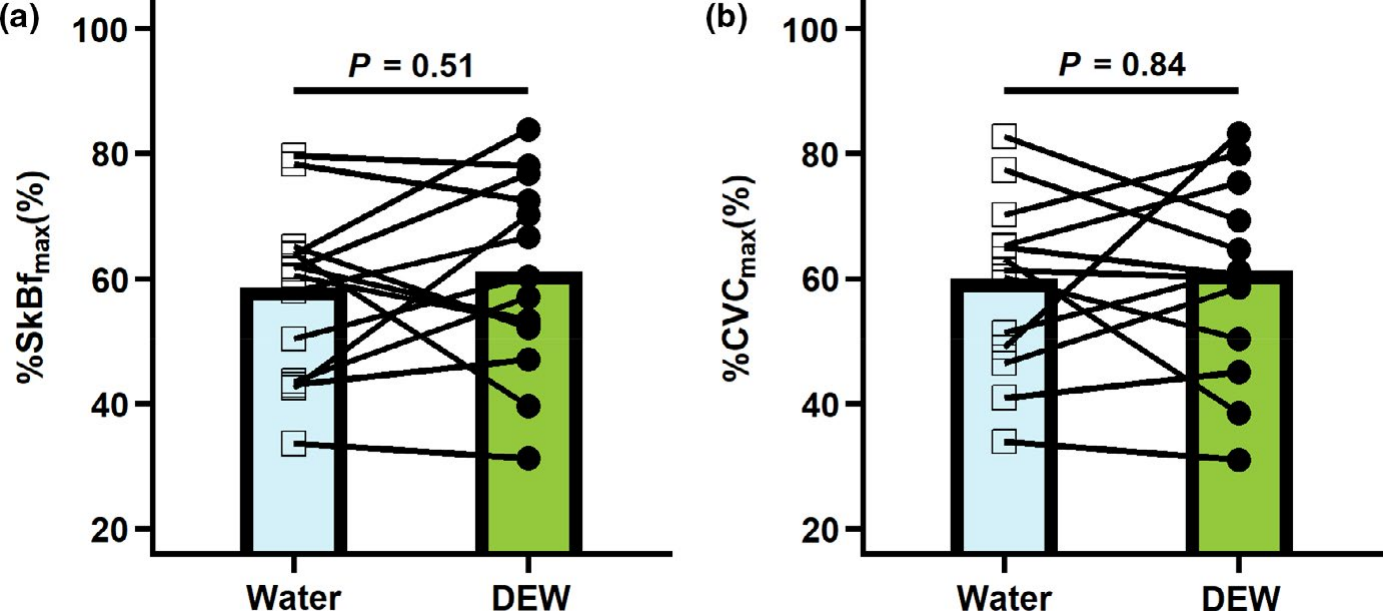
Protocol 1: Cutaneous vascular response to 500 ml fluid consumption of a caffeinated soft drink sweetened with high‐fructose corn syrup (DEW) or water (water) at the end of 40 min of local heating to 39℃. (a) Percent of max skin blood flow (%SkB_%max_) and (b) Percent of max cutaneous vascular conductance (%CVC_max_). Data are presented as means with individual responses (*n* = 14). Comparisons were made using *paired t*‐tests for DEW and water trials, *p*‐values are reported

SkBf_max_ and CVC_max_ data during local heating at 39℃ are presented in Figure 2. No differences were observed in the initial peak SkBf (%SkBf_max_ water: 55 ± 10%; %SkBf_max_ DEW: 58 ± 20%, *p* = 0.65) or CVC (%CVC_max_ water: 56 ± 13%; %CVC_max_ DEW: 59 ± 20%, *p *= 0.67). Also, no differences were observed at the end of local heating to 39℃ for SkBf (water: 185 ± 57 PU; DEW: 198 ± 79 PU, *p* = 0.43), %SkBf_max_ (Figure 2A , *p* = 0.51), CVC (water: 2.0 ± 0.6 PU/mmHg; DEW: 2.0 ± 0.8 PU/mmHg, *p* = 0.83), or %CVC_max_ (Figure 2B, *p* = 0.84). Moreover, at the end of local heating to 39℃ mean arterial pressure (water: 98 ± 11 mmHg; DEW: 100 ± 11 mmHg, *p* = 0.64), SpO_2_ (water: 97 ± 1; DEW: 97 ± 1, *p* = 0.45), and heart rate (water: 56 ± 7 bpm; DEW: 59 ± 8 bpm; *p* = 0.08) also did not differ between the DEW and water trials.

#### 3.1.2 *Protocol 2––*Effect of high‐fructose corn syrup sweetened caffeinated soft drink consumption on the vasodilatory response during local heating following ischemia‐reperfusion injury

Data collected during local heating at 44℃, following ischemia‐reperfusion are presented in Table 3. No differences were observed between the DEW and water trials in SkBf (*p* = 0.32), CVC (*p *= 0.23), mean arterial pressure (*p* = 0.21), S_p_O_2_ (*p* = 0.07), or heart rate (*p* = 0.21).

**TABLE 3:** Protocol 2 (left forearm) Heating at 44℃ following ischemia‐reperfusion and 500 ml fluid consumption

	Water	DEW	*P*‐value
SkBf (PU)	249 ± 38	237 ± 35	0.32
CVC (PU/mmHg)	2.6 ± 0.4	2.4 ± 0.5	0.22
MAP (mmHg)	95 ± 7	99 ± 11	0.21
HR (bpm)	59 ± 7	60 ± 8	0.20
S_p_O_2_ (%)	99 ± 1	99 ± 0	0.07

Note

Mean ±standard deviation.

*P*‐values from paired samples *t*‐tests are presented.

Abbreviation: SkBf, skin blood flow; CVC, cutaneous vascular conductance; PU, perfusion units (red blood cell flux); MAP, mean arterial pressure; HR, heart rate; S_p_O_2_, arterial oxygen saturation.

**FIGURE 3:**
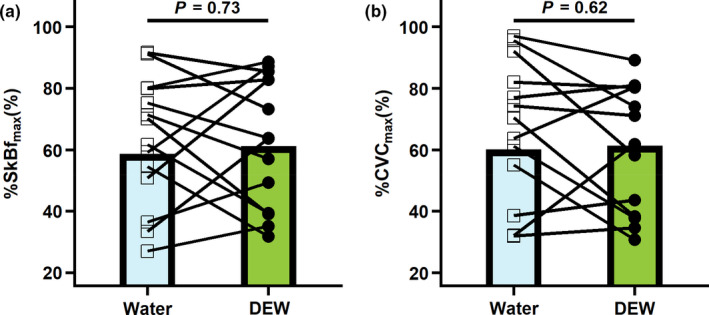
Protocol 2: Cutaneous vascular response to 500 ml fluid consumption of a caffeinated soft drink sweetened with high‐fructose corn syrup (DEW) or water (water) following ischemia‐reperfusion injury at the end of 40 min of local heating to 39℃. (a) Percent of max skin blood flow (%SkBf_max_) and (b) Percent of max cutaneous vascular conductance (%CVC_max_). Data are presented as means with individual responses (*n *= 14). Comparisons were made using paired *t*‐tests for DEW and water trials, p‐values are reported

During local heating at 39℃, no differences were observed in the initial peak SkBf (%SkBf_max_ water: 57 ± 19%; %SkBf_max_ DEW: 51 ± 20%, *p* = 0.32) or CVC (%CVC_max_ water: 59 ± 14%; %CVC_max_ DEW: 54 ± 22%, *p* = 0.48). Also, no differences were observed at the end of local heating to 39℃ for SkBf (water: 153 ± 43 PU; DEW: 151 ± 47 PU, *p* = 0.86), %SkBf_max_ (Figure 3A, *p *= 0.73), CVC (water: 1.7 ± 0.5 PU/mmHg; DEW: 1.5 ± 0.5 PU/mmHg, *p *= 0.33), %CVC_max_ (Figure 3B, *p *= 0.62), or S_p_O_2_ (water: 99 ± 0%; DEW: 99 ± 0%, *p* = 0.05). During local heating at 39℃, mean arterial pressure was 8 ± 10 mmHg [95% CI: 2–14 mmHg] higher in the DEW trial compared to the water trial (92 ± 7 mmHg vs. 100 ± 12 mmHg, *p* = 0.01). Similarly, heart rate was 5 ± 3 bpm [95% CI: 3–6 bpm] higher in the DEW trial compared to the water trial (55 ± 7 bpm vs. 60 ± 7 bpm *p* < 0.01).

**FIGURE 4:**
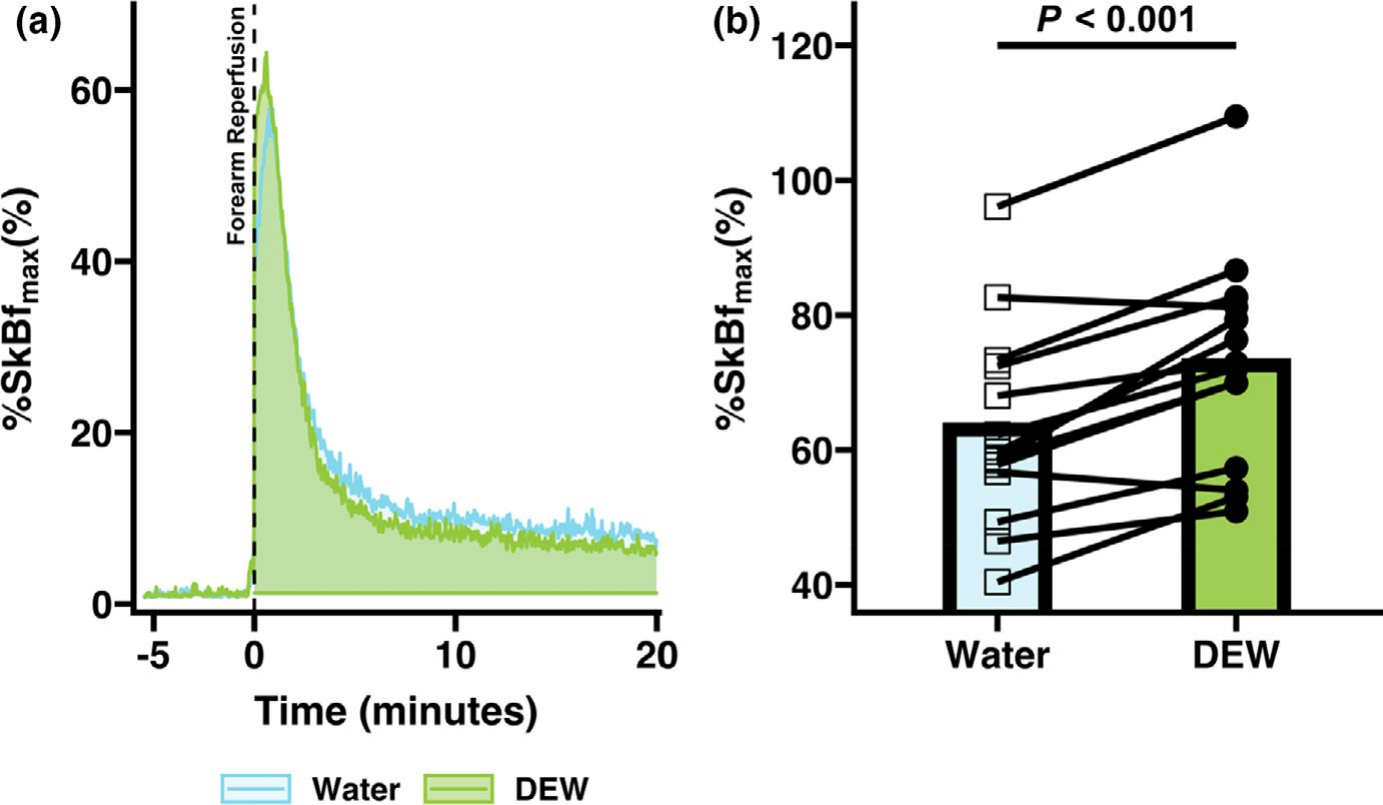
Protocol 2: Reactive hyperemia response to 500 ml fluid consumption of a caffeinated soft drink sweetened with high‐fructose corn syrup (DEW) or water (water) following 20‐min of arm ischemia. (a) Percent of max skin blood flow (%SkBf_max_) during a 20‐min reperfusion period. Data are presented as an overall group mean (*n* = 14) during the DEW and water trials. (b) Peak SkBf during reperfusion expressed as a percentage of max skin blood flow during heating at 44℃ (%SkBf_max_). Data are presented as mean with individual responses (*n* = 14). Comparisons were made using paired *t*‐tests for Dew and water trials, *p*‐values are reported

During the reperfusion period, no differences were observed in peak SkBf (water: 158 ± 49 PU; DEW: 170 ± 32 PU, *p *= 0.20) or time to peak SkBf (water: 72 ± 29 s; DEW 62 ± 24 s, *p* = 0.26). However, when expressed as %SkBf_max_ (Figure 4B), peak SkBf values during the DEW trial were shown to be greater than during the water trial (DEW: 73 ± 16%; water: 63 ± 15%, paired difference 10 ± 6%, 95% CI 6–13%, *p* < 0.01). No differences were observed in the area under the curve during the reperfusion period for SkBf (*p* = 0.42) or %SkBf_max_ (Figure 4A *p* = 0.64).

## 4 DISCUSSION

Contrary to our first hypothesis (Protocol 1), consuming 500 ml of a commercially available caffeinated soft drink sweetened with HFCS (Mountain Dew) did not attenuate the cutaneous vasodilatory response to local heating. Specifically, during local heating at 39℃, a temperature at which the vasodilatory response is ~80% nitric oxide‐dependent (Choi et al., 2014), the cutaneous microvascular vasodilator response following consumption of the caffeinated soft drink sweetened with HFCS was not different to that of water (Figure 2). Similarly, differences in the cutaneous microvascular vasodilatory response during local heating following ischemia‐reperfusion injury (Protocol 2) were not observed between the water and DEW trials (Figure 3). Collectively, our findings indicate that, following consumption of 500 ml of a commercially available caffeinated soft drink sweetened with HFCS, the skin blood flow responses to local heating with or without prior ischemia‐reperfusion injury are not different from responses observed following consumption of an equivalent volume of water.

### 4.1 Protocol 1: Effect of consumption of a caffeinated soft drink sweetened with high‐fructose corn syrup on the vasodilatory response during local skin heating (right arm)

Consumption of 500 ml of a caffeinated soft drink sweetened with HFCS causes a shift toward vasoconstriction in the kidneys (Chapman et al., 2020). In this previous study, it was concluded that the augmented vascular resistance in the renal arteries was likely contributed to by elevations in uric acid (Chapman et al., 2019, 2020). Importantly, elevations in uric acid caused by dietary fructose may reduce endothelial nitric oxide (Jia et al., 2014; Johnson et al., 2013), which is a primary driver of vasodilation (Nakagawa et al., 2005). Protocol 1 utilized the cutaneous vasculature with local heating as a model for the systemic microvasculature to non‐invasively investigate a possible mechanism of action, nitric oxide bioactivity, for examining shifts in the vasodilator–vasoconstrictor balance following consumption of 500 ml of a caffeinated soft drink sweetened with HFCS. Using this approach, the present study identified that the cutaneous microvascular response to local skin heating to 39℃ following the consumption of a caffeinated soft drink sweetened with HFCS did not differ from that observed following the intake of an equivalent volume of water (Figure 2). Since the vasodilatory response to local cutaneous heating to 39℃ is primarily a nitric oxide‐mediated event (Choi et al., 2014), it could be reasoned that consumption of 500 ml of a caffeinated soft drink sweetened with HFCS does not affect endothelial nitric oxide bioactivity. Unfortunately, this conclusion is clouded by the fact that we did not couple our assessment of the cutaneous microcirculation with intradermal microdialysis. Nevertheless, it remains possible that other mechanisms of vasodilation prevented the translation of reductions in nitric oxide bioactivity into alterations in cutaneous blood flow. For instance, in addition to the impact of uric acid on endothelial nitric oxide, hyperuricemia increases insulin resistance (Johnson et al., 2013), which could result in elevations in circulating insulin following consumption of a caffeinated soft drink sweetened with HFCS (Bloomer et al., 2016). Indeed, insulin has vasodilatory effects in the skin microvasculature, which is mediated by the nitric oxide pathway (Iredahl et al., 2013, 2016). Thus, it is possible that the resulting hyperinsulinemia (Anderson et al., 1991; Meijer et al., 2012; Schinzari et al., 2010) may have overwhelmed any fructose‐mediated reductions in endothelial nitric oxide to prevent alterations in the cutaneous vasodilatory responses to local heating. Thus, even despite our observation that consumption of a caffeinated soft drink sweetened with HFCS does not affect cutaneous vasodilation during local heating, future studies employing more mechanistic approaches should investigate the impact of dietary HFCS consumption on endothelial‐dependent and independent aspects of microvascular function.

Despite the potential mechanisms speculated on above, our findings in the cutaneous vasculature do not align with the increased vasoconstrictor tone observed in the renal vasculature following the consumption of a caffeinated soft drink sweetened with HFCS (Chapman et al., 2020). This suggests that there may be an organ‐specific effect of HFCS on vascular tone. The reasons for these potential organ‐specific effects are not immediately evident. However, a potential mechanism may be related to the role of the kidneys in fructose metabolism. For instance, excess circulating fructose is metabolized in the kidneys (Tappy & Lê, 2010), which stimulates localized uric acid production (Malik & Hu, 2015; Tappy & Lê, 2010). Moreover, the kidneys filter and excrete excess uric acid in the circulation, independent of the anatomic location of fructose metabolism. Therefore, the proximity to the production and the excretion of uric acid could explain a greater decrement in nitric oxide bioactivity in the renal vasculature compared to the skin on the forearm. Thus, following consumption of a caffeinated soft drink sweetened with HFCS the differential vascular effects between the cutaneous circulation (Figure 2), central hemodynamics (Freemas et al., 2021), and the kidneys (Chapman et al., 2020) may be partially explained by the role of the kidneys in fructose metabolism and/or uric acid handling. However, this remains to be formally investigated.

### 4.2 Protocol 2––Effect of consumption of a caffeinated soft drink sweetened with high‐fructose corn syrup on the vasodilatory response during local heating following ischemia‐reperfusion (left arm)

Tissue ischemia and subsequent reperfusion impairs endothelium‐dependent vasodilation due to the rapid production of reactive oxygen species, which can directly reduce nitric oxide bioavailability and bioactivity (Carden & Granger, 2000; Lambert et al., 2016; Wu et al., 2018), and may be exacerbated by ischemia‐reperfusion‐induced hyperuricemia (Kalogeris et al., 2012). Ischemia‐reperfusion injury has been recently shown to blunt the cutaneous vasodilatory response to local heating (McGarr et al., 2018). With this background, Protocol 2 aimed to determine if the cutaneous vasodilatory response to local heating is modified by prior consumption of a caffeinated soft drink sweetened with HFCS. Surprisingly, we did not observe any differential effects between consumption of a caffeinated soft drink sweetened with HFCS and water on the skin blood flow response to local heating following ischemia‐reperfusion injury (Figure 3). The mechanisms underlying the observed lack of differential effect on the cutaneous vasodilatory response to local heating between trials are likely the same as occurred in Protocol 1 (and detailed above), whereby the caffeinated soft drink sweetened with HFCS consumption either did not meaningfully modify endothelial nitric oxide bioactivity and/or this effect was masked by the cutaneous vasodilatory response caused by hyperinsulinemia.

An interesting finding was that consumption of a caffeinated soft drink sweetened with HFCS demonstrated a greater peak SkBf when expressed as %SkBf_max_ (Figure 4) during reperfusion. The DEW trial increase in peak SkBf is unlikely due to nitric oxide bioavailability, as NO plays a minimal role in vasodilatory peak during reactive hyperemia in the forearm (Engelke et al., (1985). 1996; Tagawa et al., 1994). Therefore, this finding suggests that other mechanisms that contribute to skin vasodilation may be involved during the consumption of a caffeinated soft drink sweetened with HFCS. Further research is required to understand mechanisms underlying this response and the potential physiological importance of this observation.

### 4.3 Experimental considerations

Both Protocol 1 and Protocol 2 took place simultaneously. This was by design to reduce participant burden and was deemed acceptable given the time course of sympathetic activation during and following forearm ischemia (Lambert et al., 2016). However, we would be remiss not to mention the possibility of interference between the two study protocols. In this light, following the completion of data collection it became clear that when viewing the amalgamated temporal data presented in Figure 1 there was an acute reduction in skin blood flow on the right arm that coincided with the commencement of reperfusion on the left forearm. This was completely unexpected, and cannot likely be explained by acute reductions in blood pressure (i.e., perfusion pressure) or increases in sympathetic activation (Lambert et al., 2016). That said, reperfusion‐induced alterations in vascular transduction (i.e., the relation between sympathetic activation and the increase in vascular resistance) cannot be excluded, although the mechanisms by which this may occur in the contralateral arm are unknown. To better understand whether this acute reduction in skin blood flow may have impacted our local heating results on the right arm (Protocol 1), we undertook a post hoc analysis to examine whether the cutaneous microvascular responses through 19 min of local heating to 39℃ (i.e., the minute before the onset of reperfusion on the right forearm) differed from those observed after 40 min of reperfusion in either trial. For comparison, we also conducted this analysis for data collected on the left forearm. These post hoc data are presented in Table 4, where it can be observed that skin blood flow and cutaneous vascular conductance did not differ between the middle and end of local heating to 39℃ on the right arm (Protocol 1) but was higher at the end of local heating versus the middle of local heating to 39℃ on the left arm (Protocol 2). Therefore, it is possible that the reperfusion period on the left forearm blunted the rise in skin blood flow to local heating on the contralateral forearm. The extent by which this may have compromised the ability to test our research hypothesis in Protocol 1 is unknown. It is notable, however, that the observed effect of reperfusion on the contralateral forearm was independent of the experimental trial. Thus, given that the water and DEW trials were conducted under the same experimental conditions, we do not believe the employed split body approach influenced the validity of our comparisons between trials. That said, we would be mistaken not to acknowledge this possibility.

**TABLE 4:** Post hoc analysis of cutaneous microvascular responses at the middle and end of local heating to 39℃ in Protocol 1 and Protocol 2

	Water		DEW		*P*‐value		
	Mid	End	Mid	End	Time	Trial	Time x Trial
Protocol 1							
Right forearm							
SkBf (PU)	190 ± 63	184 ± 59	204 ± 89	201 ± 79	0.66	0.40	0.79
CVC (PU/mmHg)	1.9 ± 0.7	1.9 ± 0.7	2.0 ± 0.9	2.0 ± 0.8	0.84	0.64	0.64
Protocol 2							
Left forearm							
SkBf (PU)	142 ± 44	154 ± 44	133 ± 47	152 ± 44	0.03	0.68	0.61
CVC(PU/mmHg)	1.5 ± 0.5	1.6 ± 0.5	1.4 ± 0.5	1.5 ± 0.5	0.05	0.40	0.88

Note

Mean ±standard deviation.

*P*‐values presented from *F*‐tests for the main effects and interaction from repeated measures ANOVA.

Abbreviation: Mid, One minute of data extracted at 19 min of local heating to 39℃. For the right forearm (Protocol 1) this was immediately before the onset of reperfusion on the left forearm; End, 1 min of data extracted at the end of local heating to 39℃. These data the same as used for the primary outcome analyses; CVC, cutaneous vascular conductance; SkBf, skin blood flow; PU, perfusion units (red blood cell flux).

While blood levels of fructose are evident 30‐min post‐consumption (Le et al., 2012), we would be remiss not to acknowledge that blood concentrations of fructose were not obtained in the current study. That said, peak circulating fructose concentrations occur 60–90 min post‐consumption following HFCS soft drink consumption and remain elevated up to 120 min post‐consumption (Le et al., 2012). Therefore, we are confident that in Protocol 1 the data reporting during heating at 39℃ coincided with the peak blood fructose levels. That said, the measurement timing of heating at 39℃ in Protocol 2 would have occurred near the end of the expected elevated blood fructose levels, which may have impacted our observations. Therefore, it is plausible that any acute effects of a caffeinated soft drink sweetened with HFCS following ischemia‐reperfusion injury could have resolved prior to obtaining the data associated with the primary outcome variables in Protocol 2.

The present study was designed based on the findings of Chapman et al. who determined cardiovascular effects of a caffeinated soft drink sweetened with HFCS consumption were caused by HFCS in young healthy adults (Chapman et al., 2019, 2020, 2021). Thus, we employed the same commercially available caffeinated soft drink sweetened with HFCS utilized previously to test our hypotheses. The downside to this approach, however, is that we cannot determine the role of individual beverage constituents on the cutaneous microvascular response to local heating. For instance, it may be that HFCS in the absence of caffeine influences the skin blood flow response to local heating. However, since caffeine does not affect forearm CVC following post‐occlusive hyperemia (Kalogeris et al., 2012) but does enhance post‐occlusive hyperemia in the fingertip skin (Engelke et al., (1985). 1996; Tagawa et al., 1994;), further research is required to elucidate how HFCS alone affects skin blood flow.

Finally, approximately half of American adults consume one or more sugar sweetened beverages on any given day (Kit et al., 2013). Thus, our young healthy participants, who consumed approximately one sugar sweetened soft drink per week, fell within the lower end of the soft drink consumption spectrum. Therefore, it is possible that our participant population may have contributed to our findings. For instance, had we recruited people who regularly consume soft drinks, perhaps we would have observed differential cutaneous hemodynamic changes between the experimental trials. Moreover, our participants arrived at the laboratory following a 2‐hr fast. Notably, fructose consumption in the fed state may augment cardiovascular risk owing to the enhanced postprandial rise in circulating triglycerides that is dependent on hepatic glycogen stores (Hengist et al., 2019). Thus, it is likely that the microvascular response to consumption of a caffeinated soft drink sweetened with HFCS is modified by hepatic glycogen status. To our knowledge, this has never been explored.

### 4.4 Perspectives

The present study demonstrates that consumption of 500 ml of a commercially available caffeinated soft drink sweetened with HFCS does not modify the cutaneous microvascular response to local heating both without and with prior ischemia‐reperfusion injury. Thus, under the experimental conditions employed herein, consumption of a caffeinated soft drink sweetened with HFCS does not acutely induce microvascular dysfunction, the latter of which is associated with a heightened cardiovascular risk (Holowatz et al., 2008). It remains plausible that chronic intake of beverages sweetened with HFCS may lead to modification of microvascular function. For example, a recent study identified that 1 week of daily consumption of a glucose sweetened beverage‐induced conduit artery endothelial dysfunction independent of changes in fasting glucose or insulin concentrations (Bock et al., 2020). Whether these findings can be translated to the cutaneous microvasculature remains to be explored. Moreover, future studies should focus on participant populations with an elevated cardiovascular risk profile, such as those with hypertension, diabetes, or obesity.

## 5 CONCLUSION

The cutaneous microvascular vasodilator response to local heating with or without prior ischemia‐reperfusion injury is not affected by acute consumption of a caffeinated soft drink sweetened with HFCS.

## CONFLICT OF INTEREST

No conflict of interest, financial or otherwise is declared by the authors.

## AUTHOR CONTRIBUTIONS

Blair D. Johnson, Stephen J. Carter, and Zachary J. Schlader contributed to study conception and design. Joel T. Greenshields, Jessica A. Freemas, Tyler B. Baker, and Zachary J. Schlader contributed to data collection activities. Joel T. Greenshields, Jason M. Keeler, and Zachary J. Schlader analyzed the data. Joel T. Greenshields, Jason M. Keeler, Jessica A. Freemas, Tyler B. Baker, Blair D. Johnson, Stephen J. Carter, Zachary J. Schlader interpreted the results. Joel T. Greenshields, Jason M. Keeler, and Zachary J. Schlader prepared the figures and drafted the manuscript. Joel T. Greenshields, Jason M. Keeler, Jessica A. Freemas, Tyler B. Baker, Blair D. Johnson, Stephen J. Carter, and Zachary J. Schlader edited and revised the manuscript, and approved the final version of the manuscript.
